# Role Clarity Among Patient Care Technicians in Saudi Arabia: Outcomes of a Structured Educational Program

**DOI:** 10.3390/healthcare14020269

**Published:** 2026-01-21

**Authors:** Nashi Masnad Alreshidi, Afaf Mufadhi Alrimali, Wadida Darwiesh Alshammari, Kristine Angeles Gonzales, Maram Nasser Alawad, Eida Habeeb Alshammari, Mohmmad Khalf Al-Shammari, Ohoud Awadh Alreshidi, Fawziah Nasser Alrashedi, Asrar Eid Alrashidi, Lueife Ali Alrashedi

**Affiliations:** 1 Nursing Executive Administration, Hail Health Cluster, Hail 55471, Saudi Arabia; 2 Academic Affairs and Training and Research Center, Hail Health Cluster, Hail 55471, Saudi Arabia; 3 Maternity and Children’s Hospital, Hail Health Cluster, Hail 55471, Saudi Arabia; 4 Erada Complex for Mental Health Hospital, Hail Health Cluster, Hail 55225, Saudi Arabia; 5 King Salman Specialist Hospital, Hail Health Cluster, Hail 55471, Saudi Arabia; 6 Cardiac Center, Hail Health Cluster, Hail 55471, Saudi Arabia; 7 Ghazalah Hospital, Hail Health Cluster, Hail 57811, Saudi Arabia

**Keywords:** patient care technicians, nursing assistants, healthcare assistants, role clarity, scope of practice, education

## Abstract

**Background:** Role clarity is a persistent challenge among Patient Care Technicians (PCTs), contributing to inconsistent task performance and safety risks. In Saudi Arabia, little is known about PCTs’ understanding of their responsibilities. This study evaluated the impact of a targeted educational program designed to improve PCTs’ role clarity, safety practices, and communication. **Methods:** A quasi-experimental pre-post study was conducted in September 2025 with 35 PCTs from the Hail Health Cluster. The one-day intervention included lectures, discussions, role-play, and case scenarios. Outcomes were measured using a validated instrument across four domains: role clarity; core clinical tasks and safety; communication and ethics; and objective knowledge. Pre-post changes were analyzed using paired *t*-tests (Cohen’s d), and subgroup differences in change scores were examined using one-way ANOVA (η^2^) in SPSS v29. **Results:** Baseline scores were lowest in objective knowledge (41.4%) and role clarity (62.8%). Post-training, total composite scores improved significantly (+10.88%, *p* < 0.001, d = 1.63), with the most significant gain in objective knowledge (+19.8%, *p* < 0.001, d = 0.99). Role clarity showed only a modest, non-significant increase (+3.98%, *p* = 0.088, d = 0.30). No demographic differences were found. **Conclusions:** Targeted training was effective in reducing knowledge gaps; however, improving role clarity may require organizational reinforcement beyond brief training.

## 1. Background

The Patient Care Technician (PCT) in Saudi Arabia is a supportive clinical role that provides direct patient care under the supervision of a registered nurse (RN) and is internationally comparable to nursing assistants/healthcare assistants [1]. Although the titles and regulatory frameworks for these roles vary widely, their core function is consistent: assisting with basic clinical procedures, daily living activities, or basic observations essential to patients’ outcomes [2]. As a measure to avoid confusion in narration within this paper, the term healthcare assistant (HCA) is used when discussing international literature, and the term Patient Care Technician (PCT) is used when referring to the Saudi context.

Globally, the utilization of HCAs has expanded in response to persistent RN shortages, rising patient acuity, and cost pressures. Although variation in job titles and regulatory frameworks complicates cross-country comparisons, there is a consistent global trend of relying on these assistive roles to maintain the continuity of patient care [2,3,4]. The scale of this workforce is significant; for example, the United States employed over 1.4 million HCAs in 2024 alone [5]. In Saudi Arabia, while supportive roles resembling PCTs have existed for years, they historically lacked formalized training or a defined scope of practice. To address this gap, the Saudi Commission for Health Specialties (SCFHS) introduced a structured, one-year PCT program through the Health Academy in 2022, which now serves as the primary pipeline for new graduates entering the healthcare workforce [6]. Currently, clinical practice within the Saudi healthcare sector is governed by a national framework requiring professional registration through the SCFHS [7]. Under this system, the SCFHS provides the competency standards and professional classifications for PCTs, while individual facilities operationalize day-to-day tasks through local policies under RN supervision [6].

Educational preparation for HCAs worldwide varies significantly, ranging from a total lack of formal training to short programs lasting only a few weeks [3,8]. The absence of consistent educational and regulatory standards has been linked to inconsistent clinical performance, professional role overlap, and increased risks to patient safety [1,2,9]. Evidence from integrative reviews further suggests that these systemic challenges are exacerbated by low nurse confidence in assistants [10,11]. Such a lack of trust inherently undermines the delegation process and serves as a significant contributing factor to missed nursing care and adverse patient outcomes [10]. When delegation frameworks are poorly articulated, assistants frequently experience uncertainty regarding their clinical responsibilities, resulting in operational confusion and compromised care delivery [12]. Furthermore, ambiguity around role boundaries is a direct barrier to effective clinical communication. Research indicates that RN perceptions of HCAs are a primary determinant of information sharing and collaboration; specifically, more positive perceptions are associated with increased communication behaviors, such as proactive seeking of feedback and higher responsiveness [8]. Conversely, a frequent misalignment exists where assistants aspire to a broader scope of responsibility than they believe their supervising nurses expect, a discrepancy that tends to stifle their participation in essential team processes [13,14]. Recent qualitative evidence from HCA-RN dyads reinforces the notion that role demarcation is often informally shaped by localized ward cultures and interpersonal negotiations rather than policy, underscoring the critical need for formal, standardized agreements on roles to mitigate professional conflict and redundant task duplication [15]. Therefore, the existence of formal job descriptions and local policies does not necessarily preclude role ambiguity, as daily clinical practice is heavily influenced by varying delegation patterns, supervisory expectations, and localized ward cultures [10,14,15].

Beyond role clarity, the safety of HCA practice relies on the reliable execution of delegated bedside tasks, meticulous infection prevention, and accurate communication and documentation. Deficits in these areas frequently lead to missed care, safety incidents, and poor team coordination [15]. As frontline providers of high-frequency bedside care, HCAs occupy a pivotal position in infection control; their daily adherence to hand hygiene, appropriate use of personal protective equipment (PPE), and compliance with isolation precautions directly impact institutional safety outcomes [16]. Furthermore, the active involvement of HCAs in clinical handovers is increasingly recognized as a vital component of patient safety, necessitating the standardization of how information is shared and documented [17]. International evidence suggests that HCAs often perform a wide range of activities, occasionally exceeding their formal training and operating with limited RN supervision, which introduces significant risks to patient safety [1]. Consequently, effective training programs must reinforce professional boundaries, specifically empowering HCAs to recognize the limits of their competence, decline tasks outside their scope, and appropriately escalate clinical concerns. Targeted safety workshops have proven effective in addressing these gaps; for example, studies show that focused training significantly improves an HCA’s ability to recognize symptoms of infections like pneumonia or urinary tract infections [18]. Similarly, even brief training sessions have been shown to increase confidence in handling clinical incidents and reduce overall adverse events [19,20]. Educational interventions that clarify job duties and boundaries have also been linked to improved role clarity and to broader team benefits such as increased confidence, better communication, and stricter adherence to safety protocols [21,22].

Establishing role clarity is a foundational requirement for effective teamwork in healthcare settings [23]. When PCTs possess a comprehensive understanding of their specific responsibilities and professional boundaries, they are better equipped to deliver safe, efficient, and patient-centered care. Accordingly, this study focuses on PCTs’ understanding of role boundaries and related practice domains. Empirical evidence on PCT roles and interventions remains remarkably limited compared to the extensive literature available on professional nurses and other healthcare providers [24]. This knowledge gap is particularly pronounced in Saudi Arabia and the broader Middle East, constraining evidence-based guidance on how to effectively translate theoretical vocational training into consistent role understanding and safe clinical practice. To address these challenges, the current study evaluates the impact of a structured one-day educational program on PCT outcomes across four domains: role clarity, safety and infection prevention, communication and ethics, and objective clinical knowledge. Additionally, the study examines how these outcomes relate to various demographic and occupational factors.

## 2. Methods

### 2.1. Study Design

This study utilized a quasi-experimental, single-group pre-test/post-test design to evaluate changes in PCT outcomes following a structured one-day educational program. The primary outcomes comprising (role clarity/boundaries, core tasks/safety and infection prevention, communication/documentation/ethics, and teamwork, and objective knowledge) were measured using a validated instrument administered immediately before and after the intervention.

### 2.2. Setting and Participants

The study was conducted in September 2025 in the Hail Health Cluster, Saudi Arabia, encompassing both secondary and tertiary hospital settings. A non-probability convenience sampling strategy was employed, targeting all eligible staff PCTs and trainees enrolled in the SCFHS program. At the time of data collection, the cluster employed 30 PCTs (21 female, 9 male); 18 participated (60%), and all eligible trainees participated (17/17, 100%). Recruitment was coordinated through Nursing Offices via email and departmental announcements for staff, and through the Academic Center in Hail for trainees. Eligibility criteria included certified PCTs working in inpatient or outpatient settings and trainees currently enrolled in the SCFHS program; PCTs on extended leave or those who declined participation were excluded.

A power analysis conducted via G*Power software (version 3.1.9.7; Heinrich Heine Universität Düsseldorf, Düsseldorf, Germany) confirmed that the final sample size (n = 35) exceeded the minimum requirement of 34 participants needed to achieve a statistical power of 0.80 for a paired-samples *t*-test with a medium effect size (d = 0.5, α = 0.05).

### 2.3. Educational Intervention

The intervention was a structured, one-day educational program developed locally within the Hail Health Cluster by nurse educators and clinical supervisors, implemented under the Nursing Executive Administration, and aligned with the SCFHS PCT curriculum to ensure professional relevance. Conducted over eight hours, the program used a blended instructional format that combined didactic teaching, small-group discussions, role-playing exercises, and interactive case-scenario simulations to promote engagement and real-world application. The program is accredited by the American Nurses Credentialing Center (ANCC), a subsidiary of the American Nurses Association and an internationally recognized body for setting standards in continuing nursing education and credentialing. ANCC accredited this program because the Nursing Executive Administration at Hail Health Cluster is an ANCC-accredited continuing nursing education provider/center, and the activity was delivered under its accreditation.

The curriculum was delivered through four core modules designed to address the full scope of PCT practice. The first module, Professional Responsibilities and Scope of Practice, defined role boundaries, delegation limits, and escalation protocols for changes in patient conditions. The second module focused on Patient Safety and Clinical Competence, covering infection control (PPE and hand hygiene), vital sign measurement, and hygiene support reinforced through practical demonstrations. In the third module, Communication, Ethics, and Teamwork, participants were trained in the Situation–Background–Assessment–Recommendation (SBAR) tool and practiced empathy through patient-centered role-play scenarios. The final module, Humanistic and Culturally Sensitive Care, addressed patient dignity and religious considerations specific to the Saudi clinical context through group case discussions. All sessions utilized printed handouts and visual presentations, with facilitators guiding scenario-based learning tailored to common ward situations to encourage critical problem-solving.

### 2.4. Data Collection Tools

Knowledge of roles and job descriptions was measured using a survey developed specifically for this study. Guided by domains highlighted in prior research on HCAs’ roles, scope boundaries, safety practices, communication and documentation standards, and professional conduct. Constructs and candidate items were mapped to established domains, then refined for coverage, clarity, and alignment with Saudi hospital practice.

The instrument consisted of four sections. The demographic section captured participant characteristics, including age, gender, employment status (employed or trainee), years of experience, area of work, orientation and refresher training, and whether the job description had been read within the previous 12 months. Section A included Likert-scale items (1 = strongly disagree to 5 = strongly agree) that assessed role clarity and boundaries (7 items), focusing on the scope of practice, tasks requiring nurse supervision, patient changes requiring escalation, and perceptions of role ambiguity. Section B examined core tasks and safety practices (7 items), including vital signs measurement, assistance with activities of daily living, infection prevention, and emergency response. Section C addressed communication, documentation, ethics, and teamwork (9 items), covering confidentiality, reporting practices, and patient interaction. Section D included (14 items) scored as true, false, or “don’t know,” which tested factual knowledge of role boundaries, patient safety, and professional conduct. The survey was administered twice: once before the educational intervention (pre-test) and again immediately after completion of the program (post-test). The survey was distributed electronically via Google Forms; for further details, the full instrument is available in Appendix A.

Face and Content validity were ensured through expert review by nursing educators. Construct validity was further supported by aligning the survey domains with the core competencies and job descriptions defined in the SCFHS PCT curriculum. The instrument was pilot-tested with 10 PCTs to assess clarity and comprehension; these participants did not take part in the educational program or the primary pre-post evaluation. Test–retest reliability was evaluated over a two-week interval using Pearson’s correlation coefficients (r) and demonstrated high stability (r = 0.84 for Role Clarity and Boundaries; r = 0.81 for Core Tasks/Safety and Infection Prevention; r = 0.89 for the Knowledge Test). Internal consistency was categorized as acceptable-to-strong, with Cronbach’s α values of 0.89 (Role Clarity and Boundaries), 0.79 (Core Tasks, Safety, and Infection Prevention), 0.73 (Communication, Documentation, Ethics, and Teamwork), and 0.75 (Knowledge Test). The overall 37-item scale yielded a composite Cronbach’s α of 0.86.

### 2.5. Data Coding and Statistical Transformation

All attitudinal and practice items were coded such that higher scores reflected clearer professional roles, safer clinical practices, and stronger communication/ethics. To ensure directional consistency, negatively phrased items, such as those regarding pressure to work beyond scope, were reverse-coded prior to aggregation. Each section’s items were averaged to yield a raw score, which was then linearly transformed to a 0–100 percentage metric based on the response range to enable direct cross-domain comparison. The knowledge test was scored objectively, with correct answers receiving one point and incorrect or “don’t know” responses receiving zero. Total scores were expressed as a percentage of the 14 items. Finally, an overall composite percentage was calculated at pre- and post-training as the mean of the four section percentages, with individual improvement computed as the percentage-point difference (Post-Pre) for each domain and the overall composite.

### 2.6. Statistical Analysis

All statistical analyses were performed using IBM SPSS Statistics, version 29.0. Descriptive statistics, including frequencies and percentages for categorical factors and means with standard deviations for continuous outcomes, summarized the sample characteristics. Section scores were computed only when all required items were present; listwise deletion was applied within each section with no data imputation. Normality of the pre–post difference scores was assessed using the Shapiro–Wilk test and was not violated (*p* > 0.05); therefore, paired-samples *t*-tests were used to compare pre- and post-training scores. Additionally, change scores (Post-Pre) were analyzed using one-way ANOVA to examine if improvements differed by participant characteristics. Statistical significance was set at α = 0.05 (two-sided), with effect sizes reported using Cohen’s d for paired *t*-tests and partial eta squared (η^2^) for ANOVA. Nonsignificant patterns were also summarized to maintain transparency.

## 3. Results

### 3.1. Sample Characteristics

Table 1 presents the demographic and professional distribution of the study cohort (n = 35). The study included 35 PCTs; the cohort was predominantly female (88.6%) and aged 20–29 years (80.0%). Employment status was balanced between staff (51.4%) and trainees (48.6%).

### 3.2. Impact of Educational Intervention on Domain Outcomes

Comparative analysis of pre- and post-test scores across domains is presented in Table 2. The program yielded a statistically significant improvement in the overall composite score, increasing from 65.99 ± 5.74% to 76.88 ± 6.46% (t = 9.65, *p* < 0.001), with a very large effect size (Cohen’s d = 1.63). Significant gains were observed across nearly all domains (*p* < 0.05), most notably in the knowledge test scores (Mean Δ 19.80%, d = 0.99) and core tasks/safety (Mean Δ 13.57%, d = 1.11). Only role clarity and boundaries did not reach statistical significance (t = 1.76, *p* = 0.088, d = 0.30).

### 3.3. Subgroup Analysis of Improvement Scores

Table 3 presents the variations in mean improvement scores across participant subgroups, as analyzed using one-way ANOVA. Overall composite improvement did not differ significantly across most participant factors (*p* > 0.05) However, a notable trend was observed for orientation status (F = 2.398, *p* = 0.107, η^2^ = 0.130): participants who reported not receiving orientation showed a higher mean improvement (20.19 percentage points) than those who received orientation (9.65) and trainees (10.95). This pattern should be interpreted cautiously, given the very small sample size of the no-orientation subgroup (n = 2).

## 4. Discussion

This study evaluated the effect of a structured one-day educational program on PCTs’ outcomes across four critical domains: role clarity and boundaries, core safety and infection prevention, communication and ethics, and objective clinical knowledge. The educational intervention yielded a significant improvement in overall knowledge, evidenced by a 10.88 percentage point increase in total composite scores (*p* < 0.001). The most pronounced gains were observed in the objective knowledge domain, which rose from a baseline of 41.43% to 61.22%. This marked improvement highlights substantial pre-existing gaps in the understanding of professional scope, safety protocols, and ethical conduct. While core clinical tasks and communication also demonstrated significant statistical growth, role clarity showed only a modest, non-significant increase (62.76% to 66.74%), representing the smallest gain across all domains. These results suggest that while a targeted educational intervention is highly effective at bridging technical knowledge gaps, improving the conceptual understanding of role boundaries remains a more persistent challenge.

Our findings align with similar pre-post intervention studies demonstrating that targeted, brief training programs can significantly enhance composite knowledge and performance scores among HCAs. For instance, structured educational initiatives in nursing home settings have previously led to greater knowledge of care and self-rated competence compared with control groups [20]. Furthermore, evidence suggests that patient safety training not only boosts HCA confidence in managing incidents but can lead to a measurable reduction in ward-level adverse events [19]. A pre/post study found that HCAs significantly improved their recognition of symptoms of Nursing home-acquired pneumonia and Urinary tract infection following targeted infection training, with gains maintained at follow-up [18]. While some literature highlights that patient safety content is often addressed superficially in HCA training [25], the substantial gains observed in our safety-related domain suggest that focused, practice-oriented programs can effectively bridge these educational gaps at the facility level. Additionally, the significant improvement in communication, documentation, and ethics observed in this study reinforces existing evidence that structured communication training, particularly using SBAR-based simulation, yields positive learning outcomes [26]. Systematic reviews indicate that such interactive methods, including the role-play and case scenarios utilized in our intervention, promote clearer and more concise information exchange [26]. Collectively, these results suggest that even relatively short, targeted programs can elevate the multi-disciplinary knowledge base of assistive personnel and improve their practical capacity for safe clinical performance.

In our sample, improvement did not vary significantly by demographic or professional characteristics, suggesting the educational intervention benefit was broadly distributed, consistent with standardized HCA training reporting comparable effects across demographic subgroups [27]. Exploratory subgroup analyses suggested a possible orientation-related pattern, with greater mean improvement among participants reporting no prior orientation than among oriented participants and trainees. Although this difference was not statistically significant, the effect size (η^2^ = 0.130) suggests a potential signal warranting further investigation. Conceptually, orientation may influence baseline readiness and familiarity with role expectations; therefore, participants without prior orientation may show larger gains after targeted training due to greater ‘room to improve [28]. However, it should be interpreted cautiously because subgroup estimates are unstable with very small cell sizes (no-orientation n = 2).

While the intervention significantly improved overall knowledge, the marginal gain in role clarity suggests that brief educational programs alone are insufficient to resolve entrenched issues related to professional identity, accountability, and delegation. Role ambiguity among PCTs stems not only from knowledge gaps but also from systemic factors, such as inconsistent supervision, vague institutional expectations, and hierarchical team dynamics. Research indicates that assistive personnel frequently navigate “informal zones of delegation,” where tasks are assigned without explicit agreement, leading to professional overlap, missed care, and interpersonal friction [10,14]. Qualitative evidence further describes delegation as a ‘double-edged sword’; while it can optimize workload, inconsistent boundaries and suboptimal supervision often compromise the quality of care processes [13,29]. Effective delegation is a complex competency requiring sophisticated clinical judgment and communication. While frameworks such as the “five rights” provide a practical structure for safe and effective delegation, their successful implementation depends heavily on clearly defined roles and supportive clinical leadership [11,30,31]. In practice, HCA responsibilities are often negotiated at the ward level, meaning role boundaries are frequently shaped by localized routines and interpersonal dynamics rather than formal policy [15]. Employment contracts typically specify job title and general duties, whereas detailed task boundaries are operationalized through job descriptions and delegation processes, and facility policies; consequently, role ambiguity often persists despite the existence of formal documentation. In our sample, most participants reported reading their job description within the past 12 months, yet role clarity/boundaries were suboptimal at baseline and improved only modestly after the intervention. This finding aligns with evidence suggesting that teamwork challenges between RNs and assistive personnel consistently center on the complex interplay of role clarity, delegation, and ward culture [11].

Role clarity represents only the initial step; its effectiveness must be sustained through robust clinical leadership and organizational reinforcement. Evidence suggests that clear role definitions, supportive supervision, and coaching, when tailored to both the assistant’s experience and the specific unit context, are directly associated with enhanced teamwork and care quality [32]. Furthermore, effective supervision serves as a protective factor against burnout and a catalyst for staff retention, whereas suboptimal supervisory support is a known driver of professional stress and turnover [33]. The performance and well-being of PCTs are also heavily influenced by broader environmental factors; heavy workloads and emotional demands, when combined with persistent role ambiguity, have been linked to significant burnout and diminished job satisfaction, particularly among younger healthcare assistants [24,34]. These systemic realities underscore the need for structured role-definition frameworks integrated into routine clinical practice. Such frameworks must be supported by continuous coaching, formalized team-based communication, and proactive leadership engagement. Ultimately, while a structured one-day educational program serves as a vital catalyst for professional awareness, long-term behavioral change requires organizational systems that actively cultivate shared clarity and mutual trust between PCTs and their supervising RN.

## 5. Implications for Practice and Policies

The findings of this study suggest that while educational interventions are vital, they must be integrated into broader institutional frameworks to achieve lasting impact. To move beyond the limitations of one-time training, the following strategic implications are proposed:

- Standardization of Scope: Formalizing PCT job descriptions and standardizing the scope of practice across the healthcare system can provide a baseline for professional accountability.
- Unit-Level Frameworks: These national standards should be supported by unit-level task frameworks that account for specific clinical environments, ensuring that delegation is both safe and context-specific under RN supervision.
- Formal Delegation Guidance: Developing structured RN-to-PCT delegation protocols, including clearly defined delegable activities, supervision requirements, and explicit documentation or escalation pathways, is essential to reduce role ambiguity.
- Interprofessional Alignment: Incorporating brief, routine RN–PCT alignment sessions into the clinical workflow can strengthen role boundaries and foster a shared understanding of team functions.
- Systemic Integration: To ensure sustainability, these role expectations and delegation standards should be embedded within formal onboarding programs and reinforced through routine audit and feedback mechanisms.

## 6. Limitations

While this study demonstrates significant short-term improvements, several limitations must be acknowledged. First, the single-group pre-post design, conducted without a control group, limits the ability to make definitive causal inferences regarding the intervention’s efficacy. Second, the brief duration of the study precluded an assessment of long-term knowledge retention or the extent to which these gains translate into sustained clinical behavior at the bedside. Furthermore, the reliance on self-reported knowledge measures may not fully reflect actual clinical practice or technical competency. Additionally, the inclusion of participants from a single health cluster may limit the generalizability of the findings to other regions or healthcare systems.

From a statistical perspective, subgroup analyses were underpowered due to very small cell sizes in certain categories, such as participants with “no orientation” or those assigned to critical care units, thereby limiting the strength of the inferences. These specific findings should therefore be viewed as hypothesis-generating rather than confirmatory.

Future research should prioritize longitudinal follow-up and the use of objective performance metrics, such as direct clinical observation or patient safety data. Moreover, developing context-tailored, multi-session training models delivered jointly to RNs and PCTs may better embed role clarity into routine, team-based practice.

## 7. Conclusions

To our knowledge, this is the first published study in Saudi Arabia to evaluate the impact of a structured educational program for PCTs. The results demonstrate that a one-day intervention can significantly enhance overall outcomes, primarily driven by substantial gains in objective clinical knowledge and core safety tasks. However, low baseline scores across several domains and only a modest, non-significant improvement in role clarity highlight the depth of pre-existing knowledge gaps and the persistent challenge of resolving role ambiguity among PCTs. While our results support continued investment in structured PCT education, they also underscore the need for complementary system-level reinforcement. This may include standardizing PCT scope and job descriptions, implementing unit-specific task frameworks and structured RN-to-PCT delegation guidance, and embedding these expectations within onboarding and routine RN–PCT alignment to reduce role ambiguity and strengthen team functioning.

## Figures and Tables

**Table 1:** Participant demographic and professional characteristics (n = 35).

Variable	Category	Count (%)
Gender	Female	31 (88.6%)
	Male	4 (11.4%)
Age group—years	20–29	28 (80.0%)
	30–39	5 (14.3%)
	40–49	2 (5.7%)
Education level	Bachelor’s degree	35 (100.0%)
Employment status	Employed	18 (51.4%)
	Trainee	17 (48.6%)
Years of experience	1–3 years	18 (51.4%)
	Does not apply (trainee)	17 (48.6%)
Area of work	Critical care unit (e.g., ICU, CCU, etc.)	2 (5.7%)
	Does not apply (trainee)	17 (48.6%)
	In-patient (e.g., Medical, Surgical, etc.)	12 (34.3%)
	Outpatient	4 (11.4%)
Orientation received	Yes	16 (45.7%)
	No	2 (5.7%)
	Does not apply (trainee)	17 (48.6%)
Refresher training in the last 12 months	Yes	9 (25.7%)
	No	9 (25.7%)
	Does not apply (trainee)	17 (48.6%)
Read job description in the last 12 months	Yes	27 (77.1%)
	No	8 (22.9%)

**Table 2:** Comparison of Pre- and Post-Training Scores Across Domains with Effect Sizes (Cohen’s d) (n = 35).

Outcome	Pre Mean ± SD	Post Mean ± SD	Mean Δ (Post-Pre)	95% CI for Δ	t (df = 34)	*p*-Value	Cohen’s d (dz)
Role clarity and boundaries (%)	62.76 ± 10.37	66.74 ± 12.33	+3.98	−0.63 to 8.59	1.76	0.088	0.30
Core tasks, safety and infection prevention (%)	79.39 ± 9.05	92.96 ± 10.16	+13.57	9.38 to 17.77	6.57	**<0.001**	1.11
Communication, documentation, ethics and teamwork (%)	80.40 ± 11.75	86.59 ± 8.74	+6.19	1.49 to 10.89	2.68	**0.011**	0.45
Knowledge test score (%)	41.43 ± 9.92	61.22 ± 15.23	+19.80	12.93 to 26.66	5.86	**<0.001**	0.99
Overall composite score (%)	65.99 ± 5.74	76.88 ± 6.46	+10.88	8.59 to 13.18	9.65	**<0.001**	1.63

Mean Δ: Represents the mean improvement calculated as the percentage-point difference (Post-test % minus Pre-test %). t (df = 34): t-statistic from the paired-samples *t*-test with 34 degrees of freedom. Cohen’s d: Effect size thresholds: 0.20 (small), 0.50 (medium), and 0.80 (large). *p*-values: Bolded values indicate statistical significance at the *p* < 0.05 level.

**Table 3:** One-Way ANOVA of Mean Improvement in Overall Composite Scores by Participant Factors and Effect Sizes (η^2^) (n = 35).

Variable	Category	Mean Improvement (Post-Pre)	ANOVA F	*p*-Value	η^2^
Gender	Female	11.143	0.401	0.531	0.012
	Male	8.879			
Age group—years	20–29	10.590	0.191	0.827	0.012
	30–39	11.488			
	40–49	13.492			
Education level	Bachelor’s degree	10.884	—	—	
Employment status	Employed	10.702	0.021	0.885	0.001
	Trainee	11.038			
Years of experience	1–3 years	10.697	0.025	0.874	0.001
	Does not apply (trainee)	11.062			
Area of work	Critical care unit (e.g., ICU/CCU)	16.071	0.503	0.683	0.046
	In-patient (Medical/Surgical)	9.755			
	Outpatient	11.409			
	Does not apply (trainee)	10.948			
Orientation received	Yes	9.654	2.398	0.107	0.130
	No	20.188			
	Does not apply (trainee)	10.948			
Refresher training (last 12 months)	Yes	12.191	0.365	0.697	0.022
	No	9.458			
	Does not apply (trainee)	10.948			
Read job description (last 12 months)	Yes	11.207	0.270	0.607	0.008
	No	9.797			

Mean Improvement: Calculated as the percentage-point difference between post- and pre-training overall composite scores. ANOVA F: F-statistic derived from one-way analysis of variance. η2 (Partial Eta Squared): Represents the proportion of variance in improvement scores explained by the participant characteristic.

## Data Availability

The data presented in this study are available on request from the corresponding author. The data are not publicly available due to ethical restrictions related to participant confidentiality.

## Appendix A

Patient Care Technician Role Clarity and Practice Survey

Demographics

1. Gender:
  - Male
  - Female
2. Age Range:
  - 20–29
  - 30–39
  - 40–49
  - 50+
3. Education level:
  - Diploma
  - Bachelor’s degree
  - Master’s degree
  - Other
4. Employment Status:
  - Employed
  - Trainee
5. Years of experience:
  - Less than 1 year
  - 1–3 years
  - 3–5 years
  - Does not apply (trainee)
6. Area of work:
  - In-patient (e.g., Medical and Surgical)
  - Critical care unit (e.g., ICU and CCU.)
  - Non-clinical area (e.g., management and administration)
  - Outpatient
  - Does not apply (trainee)
7. Orientation received:
  - Yes
  - No
  - Does not apply (trainee)
8. Refresher training in the last 12 months:
  - Yes
  - No
  - Does not apply (trainee)
9. Have you read your job description in the last 12 months?
  - Yes
  - No

5-point Likert (1 = Strongly Disagree … 5 = Strongly Agree).

Section A: Role Clarity and Boundaries

1. I know exactly which tasks are within my job description.
2. I know which tasks require an RN’s direction before I do them.
3. I understand when and how to escalate changes in a patient’s condition.
4. I know which tasks are outside my scope of practice.
5. I am often asked to do tasks not in my job description. (reverse)
6. I’m uncertain where my role ends and the nurse’s role begins. (reverse)
7. I feel pressured to perform tasks I believe are unsafe. (reverse)

Section B: Core Tasks, Safety and Infection Prevention

8. I can perform and record vital signs correctly and recognize out-of-range values.
9. I can reposition/transfer patients safely to prevent pressure injuries.
10. I apply standard precautions and hand hygiene correctly.
11. I can assist with ADLs (bathing, dressing, feeding, toileting) per policy.
12. I know immediate steps after a patient fall (safety, notify RN, document).
13. I understand skin care and basic prevention of pressure injuries.
14. I know that medication administration belongs to licensed staff.

Section C: Communication, Documentation, Ethics and Teamwork

15. I know which observations must be documented by me.
16. I understand the chain of command for reporting concerns.
17. I sometimes avoid reporting changes because I’m unsure if it’s my role. (reverse)
18. I maintain privacy/confidentiality at all times.
19. I can support culturally sensitive, respectful care.
20. I know how my role contributes to patient safety and teamwork.
21. I can describe my role to patients/families clearly.
22. I communicate with patients and families in a caring and empathetic manner.
23. I have access to resources (manuals/checklists) when uncertain.

(True/False/Don’t know). Score True/False with 1 = correct, 0 = incorrect/Don’t know.

Section D: Knowledge Test (True/False/Don’t know)

24. A Patient Care Technician may administer a pain medication independently if it is documented in the patient’s file, without consulting the nurse. (False)
25. When measuring vital signs, the nurse must be informed immediately if the systolic blood pressure is below 90 mmHg. (True)
26. It is the responsibility of a Patient Care Technician to remove a peripheral IV catheter if redness is observed at the insertion site. (False)
27. Assessing wound depth and determining its stage is the responsibility of a Patient Care Technician. (False)
28. Wearing gloves alone is sufficient; there is no need to perform hand hygiene before and after patient contact. (False)
29. Soiled linens should be carried away from the body and disposed of according to policy. (True)
30. If a patient falls, the technician should first move the patient to a safe place before informing the nurse. (False)
31. Needles must never be recapped and should be discarded directly into the sharps container(True)
32. If a patient becomes suddenly drowsy or less responsive, the technician should wait a while as the condition may improve on its own. (False)
33. When reporting changes, the ‘’S’’ and ’’B’’ components of SBAR are considered essential for a Patient Care Technician. (True)
34. Sharing details of a patient’s condition over the phone is acceptable without verifying the caller’s identity if the voice is familiar. (False)
35. Signs of pain, such as grimacing or guarding, must be documented and reported to the nurse immediately. (True)
36. Patients have the right to refuse any procedure, and the technician must respect this, inform the nurse, and document it. (True)
37. Patient privacy can be overlooked during quick tasks if the room is crowded and the work is urgent. (False)
